# Lower-Limb Power cannot be Estimated Accurately from Vertical Jump Tests

**DOI:** 10.2478/hukin-2013-0040

**Published:** 2013-10-08

**Authors:** Jean-François Tessier, Fabien-A Basset, Martin Simoneau, Normand Teasdale

**Affiliations:** 1 Faculty of Medicine - Department of Kinesiology, Laval University, Québec, Canada.; 2 Centre de recherche du CHU de Québec, Centre d’excellence sur le vieillissement de Québec, Québec, Canada.; 3 School of Human Kinetics and Recreation, Memorial University of Newfoundland, St. John’s, NL, Canada.

**Keywords:** Countermovement jump, force, validity, minimal difference

## Abstract

The countermovement jump test is often adopted to monitor lower-limb power of an individual. Despite several studies on the validity of this test, there is still a need to determine the minimal difference needed to be confident that a difference in power between two individuals is present or that a true change in the performance of an individual has occurred. In this study, power was measured from ground reaction forces and compared to that obtained from predictive equations for two groups of subjects (67 trained and 20 highly trained individuals). The height of each jump was determined with kinematic techniques. The main outcome is a large discrepancy between power calculated from ground reaction forces and that calculated from predictive equations. For the trained group, the R-square value between power and predicted power was 0.53 and the minimal difference to consider that two individuals were different was 821.7 W. For the highly trained individuals, a much larger R-square value was obtained (0.94). Despite this, the minimal difference to consider that two individuals were different was still large (689.3 W). The large minimal differences obtained raise serious concerns about using countermovement jumps for appraisal and monitoring of lower-limb power of an individual.

## Introduction

Athletes and coaches rely on physical fitness testing outcomes to monitor the workload of training periodization. Any discrepancy between the outcome of a physical fitness test and the genuine capacity of an individual could, therefore, lead to an inaccurate training prescription with the possible consequence of hindering the physical performance of an individual. Physical fitness testing procedures are designed to measure the degree of change in a performance, to predict as accurately as possible criterion scores from predictor variables, and are, therefore, fundamental measuring tools in tracking performance and monitoring training (Thomas et al., 2011).

Bosco et al. (1983) were the first to develop a field test based on the flight time of consecutive countermovement jumps to measure power in a simplest way possible. Nowadays, the countermovement jump test is routinely used to monitor the efficacy of an athlete’s conditioning program or an individual’s level of fitness (Loganet al., 2000) and several new countermovement jump tests and power estimation equations have been developed (Dugan et al., 2004; Harman et al., 1991; Johnson and Bahamonde, 1996; Sayers et al., 1999). Most of the power estimation equations were developed using ground reaction force-time data (GRF) recorded from a force platform, a technique that provides the most accurate measurements of force output during a vertical jump (Hatze, 1998; Linthorne, 2001; Sayers et al., 1999). In America, the formula developed by Sayer et al. (1999) belongs to the ACSM (American College of Sports Medicine) and CSEP (Canadian Society for Exercise Physiology) physical fitness appraisal protocols for determining power capacities of the lower body.

Although the use of predictive equations to estimate power of the lower limbs is an established practice, Hatze (1998) showed that large differences (24%) could be observed between power measured from GRF and that obtained from predictive equations. More recently, similar observations were made by Lara-Sanchez et al. (2011). As suggested by Hatze (1998), this raises some concerns about the validity of using predictive equations to estimate power. Therefore, this study aims at assessing the relationship between power measured from ground reaction forces and estimated peak power calculated from predictive equations based on the height of countermovement jumps for a group of trained and a group of highly trained athletes and to determine the minimal difference (Weir, 2005) needed to be confident that a difference between two individuals is present.

## Material and Methods

### Participants

Two sets of data were obtained from two separate experiments to constitute two distinctive groups of subjects. The first group was composed of 67 male hockey referees from the Quebec Major Junior Hockey League (age: 29 ± 7 yrs; body mass: 91.6 ± 10.4 kg; body height: 181 ± 5 cm) who participated in their overall annual physical evaluation that included the countermovement jump test. The league is one of three leagues in Canada developing players from 15 to 20 years old for professional hockey.

The second group was composed of 12 male and 8 female elite athletes (age: 23 ± 3 yrs; body mass: 76.2 ± 12.5 kg; body height: 175 ± 10 cm). Some of these athletes were world record holder, Olympians, or Canadian champions from various sports (track and field, volleyball and speed skating). Participants from both groups were familiar with the jumping technique. They all signed an informed consent form approved by Université Laval ethics committee.

### Procedures

Group 1. The participants were instructed to jump (i.e., countermovement jump) as high as they could. They were allowed to use their arms and there was no constraint on the amplitude of the countermovement but the landing of the jump needed to be on the platform. They performed 3 jumps. Each jump was separated by approximately 30 s.

Group 2. The participants performed 4 jumps with a countermovement as well. In contrast to Group 1, their arms were crossed on their chest and the amplitude of the countermovement was controlled. A flexible plastic plate was positioned at the rear of the force platform and was adjusted so that it hit the subject’s buttock at a knee angle corresponding to 90° in the eccentric phase indicating that they had to initiate the concentric phase of the jump. Before data acquisition, subjects were familiarized with this specific technique. A kinematic analysis of the knee angle that followed the data acquisition showed that all subjects generally complied with the technique as the mean knee angle at the end of the downward movement was 89.6° (SD = 6.9).

### Apparatus

Group 1. GRF parameters were recorded for all jumps with a force platform (AMTI OR6-1) fixed on the floor and surrounded by a wide wooden base. All three forces (Fx, Fy, Fz) and torques (Mx, My, Mz) were first amplified (AMTI MSA-6) and sampled at 1 kHz (12-bit A/D conversion). A reflective marker (Ligth Emitting Diode, LED) was fixed on each subject’s left greater trochanter. Two-dimensional video recordings of the jumps were taken using standard guidelines (Payton, 2008). A digital camera (Point Grey Flea) was located 3.5m from the subject, 0.9m from the floor and filmed the sagittal plane. Data collection for the digital camera and the force platform were triggered and synchronized using a frequency generator (WPI model A310-C) that also provided equidistant pulses to capture images at 50 Hz. All jumps were further analyzed by tracking displacement of the LED using MaxTraq software (Innovision Systems). GRF and kinematic data for all jumps were then imported into the Matlab environment and merged in a single file for further processing. Displacement-time signals were digitally filtered with a fourth-order Butterworth filter (10 Hz lowpass cutoff frequency with dual pass to remove the phase shift), and the maximum height of each jump was determined from the calibrated displacement-time signals of the LED placed on the greater trochanter. All force platform signals were first processed with a calibration routine and then filtered with similar parameters.

Group 2. The participants jumped on a large custom-made force platform (80 cm^2^) built using 4 strain gages (Tedea Huntleigh, model 1241 – 250 kg). All signals were first amplified (HP 8811A) before being sampled at 200 Hz (12-bit A/D conversion). The sum of all four forces was converted to obtain the resultant vertical GRF. A four-camera Selspot II systems allowed capturing the displacement of three infrared LED markers fixed on the subject’s left greater trochanter, knee lateral condyle and external malleolus. All signals were collected synchronously at 200 Hz with GRF data. As for Group 1, data were then imported into the Matlab environment for similar data processing, and the maximum height of each jump was determined from the displacement-time signals of the greater trochanter.

### Power calculation

**Figure 1:**
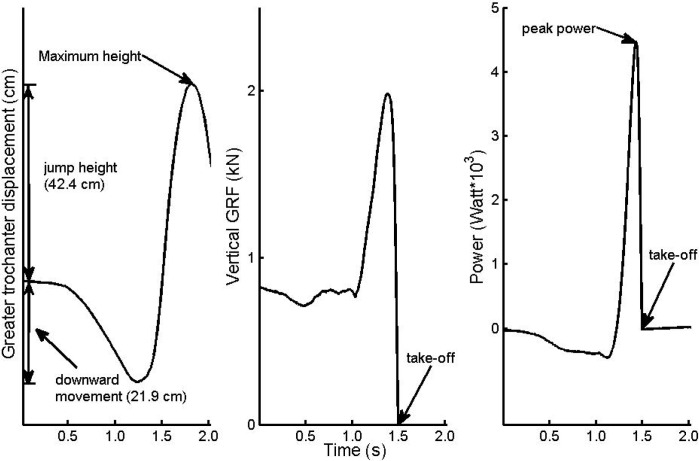
Representative jump from one subject. Displacement of the greater trochanter (left panel), vertical GRF (middle panel) and Power as a function of time

Power-time curves were obtained with a technique described by Linthorne (2001). The force-time signal was divided by the body mass of the jumper to obtain an acceleration-time signal. Then, gravity was subtracted from the acceleration-time signal and integrated (using Matlab cumtrapz function) to obtain a velocity-time signal. For each jump, the product of velocity and force resulted in the power-time curve. Peak power was obtained by identifying the highest value before take-off. Figure 1 illustrates the displacement of the greater trochanter and the force-, and power-time signals for a representative jump.

Several formulae available in literature for estimating power are based on body mass and jump height (Fox and Mathews, 1974; Harman et al., 1991; Sayers et al., 1999), and body height (Johnson and Bahamonde, 1996). In a first analysis, power estimated from all abovementioned equations was cross-correlated for data obtained for each group separately. R values exceeded 0.99 for all correlations. For the sake of brevity and because equation 2a of Sayers et al. (1999) is part of the ACSM and CSEP fitness appraisal, data is reported using this equation only (Predicted power = 60.7 • jump height (cm) + 45.3 • mass (kg) −2055). For both groups, the height of each jump was taken from the displacement of the greater trochanter (highest vertical position minus vertical position before the onset of the downward movement). Hereafter, power computed from GRF and estimated power calculated using Sayers et al. (1999) formulae are labelled power and predicted power, respectively.

Correlations, residuals, and percent difference (100*(predicted power-power)/power) between predicted power and power were computed using Statistica software. (Version 8.0, Statsoft, Inc, Tulsa, OK).

### Calculation of the minimal difference

The 3-steps approach proposed by Weir (2005) served to calculate the minimal difference (MD) in estimated peak power computed from predictive equations. MD corresponds to the minimal difference needed to be confident that a difference between two individuals is present or that a true change between two performances has occurred. First, a repeated-measures ANOVA with trial as a factor was computed to determine that the performance was not affected by fatigue or learning effects. Then, the intraclass correlation coefficient was calculated using the equation proposed by Weir (2005):

$$\mathrm{ICC}=\frac{{MS}_{S}-{MS}_{E}}{{MS}_{S}-(k-1)MS_{E}}$$

where MS_S_, MS_E_ and k were the subjects mean square, the error mean square, and the number of subjects, respectively. The standard error of measurement was then calculated

$$\mathrm{SEM}=\mathrm{SD}\times \sqrt{(1-\mathrm{ICC}).}$$

Finally, the minimal difference was obtained.

$$\mathrm{MD}=\mathrm{SEM}\times 1.96\times \sqrt{2}$$

## Results

### Power and Predicted power

Table 1 presents the correlations between power and predicted power for the two datasets. All correlations were statistically significant. For the first dataset (i.e., Group 1 - referees), the R-square value between power and predicted power (all jumps considered) was 0.53 (Standard Error of the Estimates = 512.1 W). Compared to Sayers et al. (1999) who reported an R-square value of 0.88 between power and predicted power, this R-square value was considerably smaller but the percent difference (4.4%) was similar to that reported by Sayers et al. (1999). To examine whether the smaller R-square value resulted from outliers, Studentized residual analyses were run in order to remove all jumps exceeding a value of 2.0.

**Figure 2:**
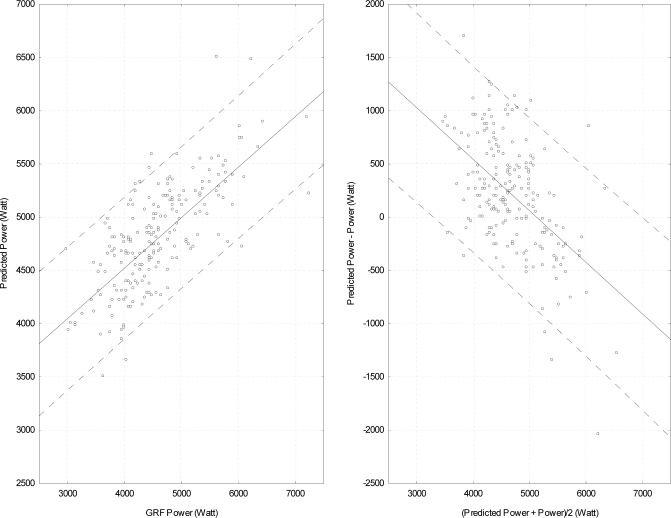
Left Panel. Relationship between power and predicted power for all jumps made by participants in Group 1. The regression line with 95% limits of agreement (broken lines) also are presented. Right panel. Bland-Altman plot of the difference (predicted power – GRF power) against average power (predicted power + GRF power/2) measurements, with 95% limits of agreement (broken lines) and regression line.

Eight jumps were removed prior to recalculating the correlation (data not shown). This procedure yielded a nearly similar R-square value (0.56). A correlation coefficient was also computed using the more standard method of selecting the highest jump of each subject (Table 1). The outcome did not differ from the previous analyses (R-square value of 0.54). Figure 2 (left panel) displays a scatter plot of power versus predicted power for all jumps of each individual for Group 1. The right panel presents a Bland-Altman plot (Bland and Altman, 1995). In this latter panel, the residuals (predicted power-power) are plotted against average of the two measurement methods (power and predicted power).

**Figure 3:**
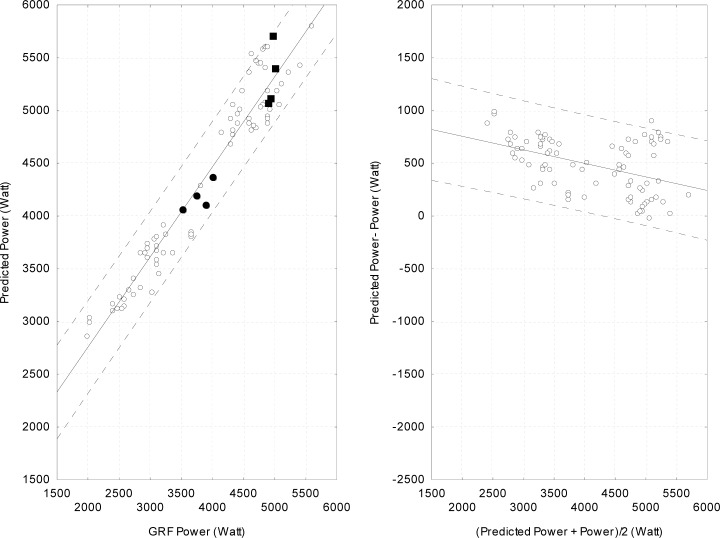
Left panel. Relationship between power and predicted power for all jumps made by participants in Group 2. The regression line with 95% limits of agreement (broken lines) also are presented. The solid squares and circles isolate jumps made by two athletes (see text in discussion). Right panel. Bland-Altman plot of the difference (predicted power – GRF power) against average power (predicted power + GRF power/2) measurements, with 95% limits of agreement (broken lines) and regression line.

The negative slope indicates that the residuals are positive for smaller power values (overestimation) and negative (underestimation) for higher power values. Also, large residuals are observed throughout the range of power values. One could postulate that the low R-square value and the variability relate to individuals tested. Although the test was part of a global evaluation procedure for selection purposes of a relatively homogeneous group, it could be argued that the referees were not familiar enough with the jumping technique or that the athletic potential and level of fitness varied widely across referees. This was not the case for individuals in Group 2 as all of them were highly trained athletes familiar with the jumping technique. Compared to Group 1, the R-square value obtained, when all jumps were considered, was higher (0.94; percent difference = 12.5%) (Table 1). As for Group 1, a Studentized residual analysis was computed and two trials were removed prior to recalculating the R-square value (0.94). The analysis with the highest jump of each subject yielded a similar R-square value (0.95). Hence, none of the abovementioned analyses (neither residual analyses nor using the highest jump) improved the R-square value significantly compared to the analysis including all jumps of each individual. Figure 3 (left panel) presents a scatter plot of the power vs. predicted power for all jumps of athletes. For comparison purposes, the axis for the Bland-Altman plot (Figure 3, right panel) uses the same scaling than that of Figure 2. As for Group 1, the slope of the relation is negative, but the mean residual is positive indicating that, generally, predicted values were overestimated for Group 2. This may reflect that subjects in this group were highly familiar with the jumping technique and have learned to optimize the height of their jump. Also, compared to Group 1, a smaller range of residuals is observed.

Minimal Difference (MD)

As mentioned above, the key question being asked relates to the smallest difference needed to be confident that a difference between two individuals is present. Following a training program aiming at increasing power, a similar question would be asked: What is the smallest difference to be confident that a difference between baseline and post-training performance is a true difference. For Group 1, results from a repeated-measures ANOVA with a trial as a factor yielded a non-significant effect of the trial (F(2,132)= 1.96, p > 0.05). The ICC and SEM were 0.87 and 296.4 W, respectively. Finally, the MD value for the predicted power was 821.7 W. Similar calculations were done for Group 2. The ANOVA yielded a significant effect of trial (F(3, 57)= 2.81, p < 0.05). A comparison of means (Tukey test) revealed that power for Trial 1 was smaller than that for the last three trials (p < 0.05). As suggested by Weir (2005), data for the first trial were removed from the analysis. The new ANOVA yielded a non-significant effect of trial (F(2, 38)= 0.25, p > 0.05), and only the last three trials were used for computing the MD. The ICC and SEM were 0.97 and 248.7 W, respectively. The MD value was 689.3 W.

## Discussion

Power measured from GRF is accepted as a reliable indicator of total muscular effort that ascertains whole-body acceleration (Hatze, 1998; Zatsiorsky, 2002). Other means for measuring force and power are actual estimation based on predictive equations. The present study was designed to compare power measured from GRF to that obtained from predictive equations and to compute the minimal difference needed to be confident that a true difference between two individuals existed. The main outcome is a large discrepancy between power calculated from GRF and predictive equations and large minimal differences for both groups that were tested. This was observed despite the fact that a large R-square value between power measured from GRF and predicted power (for instance, R-square = 0.94 for Group 2) was obtained and that percent difference between predicted power and power was within a range of values previously reported. For instance, a percent difference of 5% was reported by Sayers et al. (1999) for equation 2b. More recently, Lara-Sanchez et al. (2011) reported a value of 11% for the same equation. Previously, Hatze (1998) reported a difference of 24% with predicted values from the Bosco’s equation. Values as high as 72% have been reported by Sayers et al. (1999) for an equation proposed by Lewis (available in Fox and Mathews, 1974, p. 257–258). In the present study, percent differences of 4.4% and 12.5% were obtained for Group 1 and 2, respectively.

Large residuals were observed for both groups and the minimal difference was large for both sets of data (821.7 W and 689.3 W for Group 1 and 2, respectively). This raises serious concerns with regard to using predictive equations for monitoring training progress of an individual. For instance, a recent meta-analysis suggests that plyometry training improves vertical jump height (squat jump protocol) by 4.7% (Markovic, 2007), that is, mean increased power of 218 W. For an individual weighting 75 kg, this corresponds to an increase of 3.6 cm in jump height. An analysis of the residuals obtained in the present study allows appreciating this value. For Group 1, 84.6% of the jumps (170 out of 201 jumps) showed a residual greater than 250 W. For Group 2, 42.5% of the jumps (34 out of 80 jumps) showed a residual greater than 250 W (with one jump showing a residual of 531 W). As mentioned above, these large residuals are not unique to this study (as an example, similar large residuals can be inferred from Figure 1 of Sayers et al. (1999)). Using the highest of three jumps did not reduce the size of the residuals. Clearly, the minimal difference obtained for both datasets was too large for suggesting that any training leading to a 218 W improvement in power would be significant.

Another way to express these large minimal differences is to examine specific power values for a participant. Solid squares presented in Figure 3 depict the data of an athlete who produced a 119 W variation in power (calculated from GRF parameters). However, despite this small variation between four different jumps, a variation of 647 W is observed in predicted power between the four jumps. A positive residual of 700 W is also noted for one jump. The solid circles in Figure 3 depict data of another athlete displaying a different pattern. For this athlete, while a variation of 486 W across the four jumps was measured with GRF, a smaller variation of 314 W was obtained with the equation. These mismatches between power and predicted power could lead to an inaccurate appraisal and training prescription.

There are several possible sources to explain the large minimal differences and the residuals between power and predicted power. First, the suggestion that the height of a jump is an accurate predictor of power originates from mechanics and the underlying hypothesis that all segments are rigid bodies. Humans are multi-joint deformable bodies with several possibilities for energy to dissipate. Hatze (1998) demonstrated in a detailed biomechanical analysis of countermovement jumps that at least 3% of the total power is lost in the form of internal segmental energy flows and nonvertical power components. The precise timing and coordination of muscle action (Bobbert and Van Soest, 1994; Pereira et al., 2008) and upper body movements (Lees et al., 2004) also are key factors for optimizing the height of the jump and high-level biomechanical analysis is required to tease apart these different contributions. For instance, Lees et al. (2004) showed that using the arms allows increasing the height and velocity of the center of mass at take-off, thus leading to a greater jump height. At least, for Group 1, variability in the technique of using the arms could have added some within- and between-subject variability in jump height (Floría and Harrison, 2012; Lees et al., 2004). Finally, variable GRF and a nonlinear increase of velocity during the propulsion phase also can account for the discrepancies between power and predicted power (Hatze, 1998; Lees et al., 2004). Altogether, these various sources add up to generate significant errors between predicted power and power. The large minimal differences that were obtained in the present study are in accordance with Hatze’s (1998) suggestion that jumping ergometers and predictive equations cannot be considered reliable to measure power. This suggests vertical jump tests are of a little practical use for the assessment and monitoring of an individual’s power or for comparing two individuals. Using predictive equations to estimate power may lead to gross over or under-estimation of power and may result in prescribing inaccurate training intensities that could lead to detrimental effects on sports performance and motivation in highly trained athletes (for instance, through providing misleading feedback about the result of a training program).

When monitoring power in elite athletes, very sensitive measuring devices are required to detect small margins in performance improvement. Unfortunately, the various marketed apparatuses that estimate power from jump height or flight time lack reliability and validity. They should not be used for sports in which power determines performance (e.g., skiing, skating, running, sprinting, etc.). These devices are still valid to monitor jump height. Coaches and trainers must, therefore, take into account these limitations. Future investigations should aim at developing power ergometers using innovative designs that are more sport specific.

In conclusion, despite the broad use of the vertical jump test to estimate power, residuals observed between power calculated from GRF and power obtained from predictive regression equations and the large minimal differences observed preclude the use of these equations for monitoring an individual’s power. The vertical jump test provides information about the ability of an individual to jump vertically, and the height of the jump should not be interpreted (through a predictive equation based on the height or the flight time of a jump or a series of jumps) as a true measure of the power an individual can generate but more as a measure of the jumping ability of an individual.

## Figures and Tables

**Table 1:** Means, standard deviations and correlations between power and predicted power for Group 1 and Group 2. All correlations are significant at p < 0.05. SEE = Standard error of estimate. M Residual = Mean residual. SE Residual = Standard error of the residual. For each group, results are presented first for all jumps made by all individuals and then for the highest jump of each individual.

	Power (W)									
	M	SD		M	SD	r	r^2^	SEE	M Residual	SE Residual
**Group 1** N=201	4586.4	749.2	Predicted power	4799.2	489.5	0.73	0.53	512.1	395.38	22.19
highest jump N=67	4641.3	729.1	Predicted power	4933.8	472.3	0.74	0.54	495.9	380.90	36.38
**Group 2** N=80	3863.4	974.6	Predicted power	4349.1	858.8	0.97	0.94	243.7	205.83	13.65
highest jump N=20	3945.5	1016.9	Predicted power	4517.7	903.3	0.98	0.95	230.7	179.11	27.85
